# Comparison of the 36-Item WHODAS 2.0 Functional Assessment in Older Adults by Face-to-Face or Telephone Interviews: A Randomised Crossover Study

**DOI:** 10.3390/jcm14227902

**Published:** 2025-11-07

**Authors:** Agnieszka Sozańska, Bernard Sozański, Anna Wilmowska-Pietruszyńska, Zofia Dzięcioł-Anikiej, Magdalena Hagner-Derengowska, Agnieszka Wiśniowska-Szurlej

**Affiliations:** 1 Faculty of Health Sciences and Psychology, Collegium Medicum, University of Rzeszow, Rejtana16C, 35-959 Rzeszow, Poland; agwisniowska@ur.edu.pl; 2 Inter-University Center for Disability Studies, University of Rzeszow, Rejtana16C, 35-959 Rzeszow, Poland; bsozanski@ur.edu.pl; 3 Health, Functioning and Disability Research Laboratory, University Research and Development Center in Health Sciences, Warzywna 1a, 35-310 Rzeszów, Poland; 4 Faculty of Medicine, Collegium Medicum, University of Rzeszow, Rejtana16C, 35-959 Rzeszow, Poland; 5 Faculty of Medicine, Lazarski University, Świeradowska Street 43, 02-662 Warsaw, Poland; anna.wilmowska@autograf.pl; 6 Inter-University Center for Disability Studies, Lazarski University, Świeradowska Street 43, 02-662 Warsaw, Poland; 7 Faculty of Health Sciences, Department of Rehabilitation, Clinical Research Center (CBK), Medical University of Bialystok, Kilinskiego 1 Street Bialystok, 15-089 Bialystok, Poland; zofia.dzieciol-anikiej@umb.edu.pl; 8 Sports Research Center, Nicolaus Copernicus University, Lwowska Sreet 1, 87-100 Toruń, Poland; m.hagner-derengowska@umk.pl

**Keywords:** older adults, disability assessment, WHODAS 2.0, telephone interview, face-to-face interview, reliability, telemedicine

## Abstract

**Background/Objectives:** With the expansion of telemedicine, it is essential to determine whether the telephone administration of validated disability measures yields results equivalent to face-to-face interviews in older adults. We aimed to compare the agreement between face-to-face and telephone administration of the 36-item WHODAS 2.0 in a community-dwelling population aged ≥60 years. **Methods:** The study was a randomised, open-label, cross-over design involving 239 people aged ≥60 from south-eastern Poland. Participants were randomly assigned to two groups: G1 (face-to-face interview first, then telephone interview) and G2 (in the reverse order). The interval between interviews was an average of 14.3 ± 1.3 days. The full version of WHODAS 2.0 (excluding domain D5.2) was used. Cohen’s kappa coefficient (κ), intraclass correlation coefficient (ICC) and Bland–Altman analysis were used to assess the consistency of the results. **Results:** A total of 203 individuals completed both surveys. The agreement between responses for individual items ranged from 85.71% to 98.03% (κ = 0.788–0.947). The WHODAS 2.0 total score showed very high agreement between face-to-face and telephone interviews (ICC = 0.986). All domains showed high or very high agreement (ICC = 0.953–0.967). Bland–Altman analysis confirmed high agreement—only 5.42% of results fell outside the 95% limits of agreement. **Conclusions:** Telephone interviews are a reliable alternative to face-to-face interviews when using the 36-item WHODAS 2.0 in older adults. Both methods provide comparable and reliable results, confirming the usefulness of telephone interviews in clinical practice and screening, especially when access to in-person healthcare is limited.

## 1. Introduction

The ageing of populations worldwide is a significant demographic trend, resulting primarily from increasing life expectancy and declining fertility rates [1]. In Poland, at the end of 2023, people aged 60 and over accounted for 26.3% of the total population. The old-age dependency ratio was 30.9. According to demographic projections, the number of older adults in Poland is expected to grow steadily until 2060, reaching 38.3% of the total population [2].

According to data from the World Health Organisation (WHO), nearly 16% of the global population experiences disability, with the highest percentage among older adults [3]. According to a report by the Central Statistical Office (GUS), 45.1% of older adults in Poland had a limited ability to perform activities due to health problems lasting at least the past 6 months in 2023 [2].

With age, the risk of chronic diseases, social isolation, reduced activity, functional limitations and disability increases. Multimorbidity is associated with faster progression of disability in older adults [4,5,6]. Maintaining mental and physical fitness, activity and participation in social life is essential for maintaining independence in performing daily activities, good quality of life and reducing the risk of hospitalisation, institutionalisation and mortality [7,8,9].

The assessment of older adults’ functioning is a key indicator for evaluating the risk of disability onset and progression [10,11,12]. This assessment provides important information on the extent of the problem in performing various activities, the level of functional difficulty and disability, and estimates the risk of frailty and dependence. It is also an important clinical measure. Functional assessment improves diagnostic accuracy, treatment selection, prediction of clinical outcomes, and monitoring of the health status of older patients [13,14].

In this context, an important tool is the WHO Disability Assessment Schedule 2.0 (WHODAS 2.0)—a universal, standardised questionnaire assessing the level of disability and difficulties in activity and participation, in accordance with the ICF classification [15,16]. This tool has high measurement accuracy and reliability [17] and is widely used in various settings, including the older adult population [18,19]. WHODAS 2.0 is available in two versions: full (36 items) and short (12 items). The 36-item version provides information about the overall level of disability and the level of disability in six domains of functioning. The 36-item version is detailed, accurate and can be used to fully characterise the level of functioning, while the 12-item version is quicker and suitable for brief assessments or screening [15]. Due to its simplicity and accessibility, both versions of the tool can be effectively used to identify individuals with functional problems and to plan appropriate interventions to prevent loss of fitness.

In the era of telemedicine tools and healthcare digitisation, the ability to assess the functional status of older adults using telephone interviews is becoming increasingly important. This form of communication is fast, economical and acceptable to older adults. However, despite growing interest in this form of data collection, there are a limited number of studies evaluating the reliability of screening and clinical tools used in telephone interviews. None of these studies strictly assess the validity of WHODAS 2.0 measurements using face-to-face and telephone interviews.

To date, no studies have been published comparing the consistency of assessments conducted using the 36-item WHODAS 2.0 in face-to-face and telephone interviews in the older adult population. Therefore, the aim of this study was to compare the consistency of results obtained using the 36-item WHODAS 2.0 questionnaire in two forms—face-to-face and telephone interviews—among older adults.

## 2. Materials and Methods

### 2.1. Study Design

Randomised, open-label, crossover study with two study periods (sequences AB/BA), comprising four assessment points (two in each sequence). The chosen cross-over study design, where each participant serves as their own control, reduces inter-individual variability and increases the precision of comparisons between assessment methods. This allows for greater statistical power with fewer participants and better control of confounding factors [18,19]. The study design was developed in accordance with COSMIN guidelines, which ensured high methodological quality and minimised systematic error in the assessment of the tool’s reliability [20].

### 2.2. Participants and Setting

The study was conducted among older adults living in the community in south-eastern Poland, using the 36-item WHODAS 2.0.

The study was conducted using a diagnostic survey method. Participants were recruited from randomly selected health centres in the Podkarpackie Province. Recruitment of participants was carried out in cooperation with primary care physicians in selected centres. In each centre, patients meeting the inclusion criteria were randomly selected from among those reporting for follow-up or preventive visits. Those interested in participating received verbal and written information about the study and then signed an informed consent form. To ensure representativeness, both rural and urban residents were included. Participants were scheduled for a specific date for a face-to-face or telephone interview, according to a randomly assigned study sequence.

The inclusion criteria were: age 60 years and older, normal cognitive function (Abbreviated Mental Test Score (AMTS) > 6 points), and informed consent to participate in the study. The exclusion criteria were: staying in a nursing home, significant hearing, speech, or visual impairments, acute or unstable medical conditions, communication barriers (e.g., language difficulties or lack of telephone access).

The study was conducted between 15 June 2025 and 15 July 2025. Participants were randomly assigned to two groups (G1 and G2) using a computer-generated randomisation list created in Microsoft Excel (RAND function) by an independent researcher who was not involved in recruitment or assessment. The allocation sequence was applied consecutively according to the order of participant enrolment. This approach ensured an unbiased and transparent randomisation process. Each participant underwent two measurements using the WHODAS 2.0 questionnaire (36-item version). In group G1, the first interview was conducted face-to-face (A) and the second by telephone (B). In group G2, the reverse order was used—participants first responded by telephone (B) and then face-to-face (A). The average interval between the two assessments was 14.3 days (SD = 1.3).

Each interview lasted approximately 25–35 min. At the beginning of each measurement, the interviewer reminded the participant that the answers should refer to the situation in the last 30 days.

During the second assessment, an additional check was made to ensure that no adverse events or situations had occurred between interviews that could significantly affect the participant’s physical or mental health, such as hospitalisation, injury, exacerbation of a chronic disease, or severe psychological stress. If such circumstances were found, the participant was excluded from the comparative analysis to avoid misinterpretation of any changes in the results.

To minimise the risk of systematic error (bias) and in accordance with COSMIN guidelines, independent interviewers participated in the study—each of the two forms of interview (face-to-face and telephone) was conducted by a different person, and the interviewers did not have access to the results of the previous measurement.

All researchers underwent training covering the principles of conducting interviews in accordance with WHO recommendations for the use of the WHODAS 2.0 questionnaire, which ensured standardisation of the procedure and high repeatability of the measurement.

### 2.3. Measurement

The tool assessed in the study was the 36-item World Health Organisation Disability Assessment Schedule 2.0 (WHODAS 2.0). WHODAS 2.0 is a disability assessment tool developed based on the codes of the International Classification of Functioning, Disability and Health (ICF). The WHODAS 2.0 tool was developed by the World Health Organisation and is recommended as a standard instrument for assessing disability [21]. Its Polish version has been validated in the older adult population, confirming its high psychometric properties [22].

The extended version of WHODAS 2.0 assesses disability in six domains: Cognition (D1), Mobility (D2), Self-care (D3), Getting along (D4), Life activities—household (D5.1) and Life activities—work/school (5.2), Participation (D6). Life activities (5.2)—work/school were not analysed in this study because the respondents were retired, and the vast majority were no longer working. According to the instructions for calculating the 36-item WHODAS 2.0, it is possible to exclude domain 5.2 from the analysis [21].

The WHODAS 2.0 response scale is a 5-point scale, where 1 = no problem, 2 = mild problem, 3 = moderate problem, 4 = severe problem, 5 = complete problem. The results were converted to a scale from 0 to 100. The degree of disability is presented on a qualitative scale according to the ICF, from 0 to 4% (no disability), from 5 to 24% (mild disability), from 25 to 49% (moderate disability), from 50 to 95% (severe disability) and from 96 to 100% (severe disability) [21].

The Abbreviated Mental Test Score (AMTS) questionnaire was used to assess cognitive status, which was one of the criteria for inclusion or exclusion from the study [23].

In addition, the study used an interview questionnaire that included personal details and questions about health status and subjective assessment of quality of life. In the first stage of the study, socio-economic data were collected, such as age, gender, place of residence, marital status, number of people in the household, and basic health information, including the presence of chronic diseases confirmed by a doctor and the number of medications taken daily. In both the first and second stages of the study, pain intensity was assessed using a numerical rating scale (NRS) and a self-assessment of quality of life (QOL) was conducted based on the question: “How would you rate your quality of life?” with a five-point response scale (“very good”, “good”, “neither good nor bad”, “bad”, “very bad”). In the second round of the study, participants were also asked about any difficult life events that may have occurred since the first measurement, such as the death of a loved one or a serious deterioration in health (e.g., an accident with injury, diagnosis of a serious illness) that could significantly affect their mental or physical condition. Individuals who had experienced such events were excluded from further analysis.

### 2.4. Sample Size

The sample size was estimated using Statistica 13 software (TIBCO Software Inc., 2017; Statistica—data analysis system, version 13, http://statistica.io). It was assumed that the percentage of participants for whom the differences between the overall disability scores obtained in the 36-item WHODAS 2.0 questionnaire using two different methods would fall within the 95% confidence interval according to the Bland–Altman method would be at least 95%. With an acceptable error of 4% and a confidence level of 99%, the minimum number of participants needed to conduct the study was 196. Taking into account possible data gaps or participant loss of up to 15%, the final sample size was estimated at 231 people.

To further secure the required statistical power and compensate for potential unforeseen exclusions due to adverse events or lack of follow-up, a slight oversampling was applied. Consequently, 239 participants were recruited, representing a 3.5% increase over the initially planned sample size. This conservative approach ensured the completeness and robustness of the final analyses.

### 2.5. Statistical Analysis

**Figure 1:**
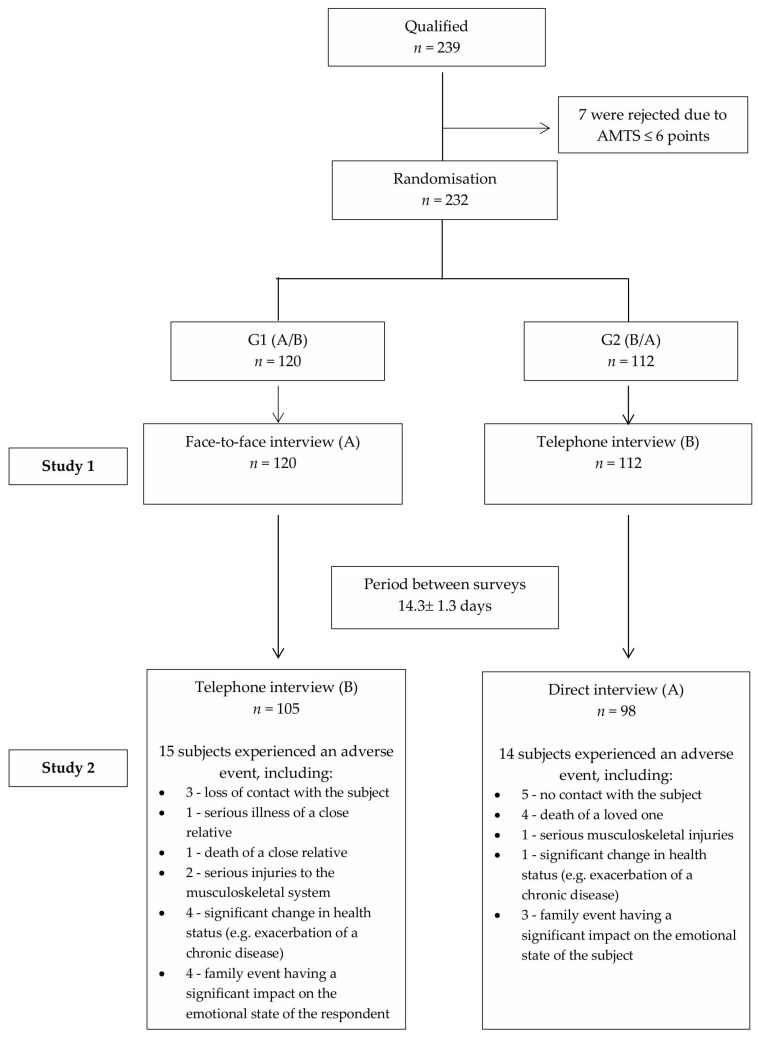
Flow diagram of the study population.

A block diagram illustrating the recruitment of the study group was developed (Figure 1). For sociodemographic variables characterising the study participants, the number and percentage (%) for categorical variables or the mean ± standard deviation (SD) and quartiles for quantitative variables were given. The chi-square test was used to assess the significance of differences between sociodemographic characteristics in the study groups for categorical variables, while the Mann–Whitney U test was used for quantitative variables. The normality of the distribution of quantitative variables was tested using the Shapiro–Wilk test. A significance level of *p* = 0.05 was adopted.

Cohen’s kappa coefficient (κ) was used to assess the consistency of the results obtained for individual questions. The level of agreement was interpreted as: 0.00–0.20—none, 0.21–0.39—minimal, 0.40–0.59—weak, 0.60–0.79—moderate, 0.80–0.90—strong, above 0.90—almost perfect [24]. The consistency of quantitative variable assessments was evaluated using the intraclass correlation coefficient (ICC). The ICC3 coefficient (according to the Shrout and Fleiss classification) was used. The level of agreement was interpreted as: below 0.50—poor agreement, 0.50–0.75—moderate agreement, 0.75–0.90—good agreement, 0.90–1.00—very good agreement [25]. The Bland–Altman method was used to assess the agreement of the summary results between measurements obtained by the two methods [26]. The normality of the distribution of differences was checked using the Shapiro–Wilk test. The following measures of agreement were calculated: repeatability coefficient (RC), variation coefficient (VC), limits of agreement (LOA) and Bland–Altman Index (BA). Methods were considered consistent if the percentage of individuals outside the range of agreement did not exceed 5% [27].

The analyses were performed using TIBCO Software Inc. (2017), Statistica (data analysis software system), version 13. http://statistica.io and R software, version 4.5.0 [28].

### 2.6. Ethics Approval

The study design was approved by the Bioethics Committee of the University of Rzeszów (Resolution No. 053/06/2025/A). In accordance with the guidelines of the Declaration of Helsinki, all participants were informed about the purpose and course of the study and then obtained their written consent to participate. Participants were also informed of their right to withdraw from the study at any stage without having to give a reason.

## 3. Results

### Characteristics of the Study Population

A total of 239 participants were selected and randomly assigned to the study (Figure 1). Based on the AMTS test, 232 individuals who scored at least 7 points were qualified for the first study. These individuals were randomly assigned to groups G1 and G2. A total of 203 individuals (87.50% of the first study participants) were included in the analysis. All questionnaires intended for analysis were complete. The differences in the number of questionnaires included in the final analysis were due to the exclusion of results from individuals who experienced an adverse random event between surveys that had a significant impact on their physical and/or emotional assessment, as well as a lack of contact with the respondent. The percentage of individuals whose results were not analysed was identical in both groups (group 1: *n* = 15/120, 12.50%; group 2: *n* = 14/112, 12.50%). The interval between the two surveys was 14.3 ± 1.3 days.

The average age in the study population was 73.48 ± 6.95 years, with 73.65 ± 7.18 years in the first group and 73.31 ± 6.74 years in the second group. The study group was characterised by a higher percentage of women (125; 61.58%). Most of the study population had vocational or lower education (47.78%) and lived in rural areas (58.62%). The mean number of diseases was 4.94 ± 2.92, and the average pain intensity on the NRS was 3.76 ± 2.16. The AMTS in the study population was 9.28 ± 0.99 points.

The slight asymmetry in group sizes resulted from the use of block randomisation with blocks of varying sizes, which was intended to maintain random allocation while ensuring flexibility in the recruitment process. The uneven distribution did not affect the reliability of the comparisons, as the two groups did not differ significantly in terms of socio-demographic or clinical characteristics (Table 1).

For the 32 questions analysed in the WHODAS 2.0 questionnaire, the percentage of consistent responses given by respondents during the first and second surveys ranged from 85.71% (Question D6.4) to 98.03% (Question D3.3). Cohen’s kappa coefficient values ranged from 0.788 (Question D6.4) to 0.947 (Question D1.1). The results indicated an almost perfect level of agreement in the case of 9 questions, a strong level in the case of 19 questions, and a moderate level in the case of 4 questions (Table 2).

The agreement between face-to-face and telephone interviews for the total score of the 32-item WHODAS 2.0 was assessed using the intraclass correlation coefficient and the Bland–Altman method. In the case of the total score of the 32-item WHODAS 2.0, the correlation coefficient (ICC = 0.986) indicates a very high consistency of the results obtained (Table 3).

**Figure 2:**
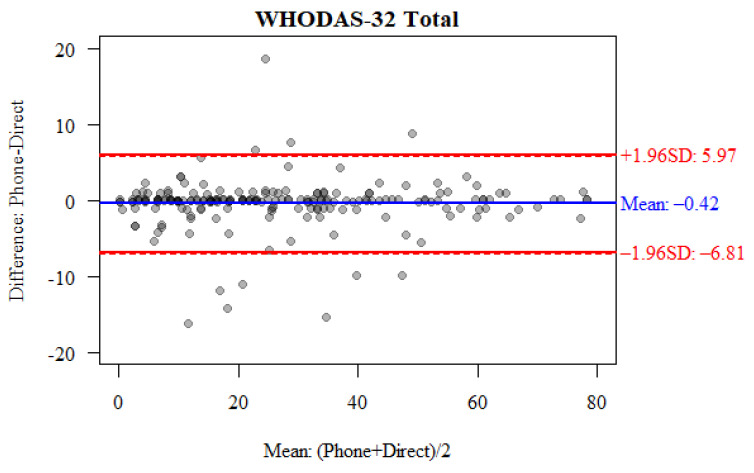
Bland–Altman plot comparing the total score of the 32-item WHODAS 2.0 for face-to-face and telephone interviews.

The consistency of the results obtained for the total score of the 32-item WHODAS 2.0 was additionally confirmed using the Bland–Altman method. The results of 5.42% of the respondents did not fall within the 95% consistency range, which in this case was from −6.81 to 5.97 points (Figure 2).

In a similar manner, the consistency of face-to-face and telephone interviews was assessed for the results obtained in individual subscales.

For all subscales of the 36-item WHODAS 2.0 (excluding subscale 5.2), ICC correlation coefficients ranging from 0.953 (Do5.1) to 0.967 (Do3) indicate a very high consistency of the results obtained (Table 4).

**Figure 3:**
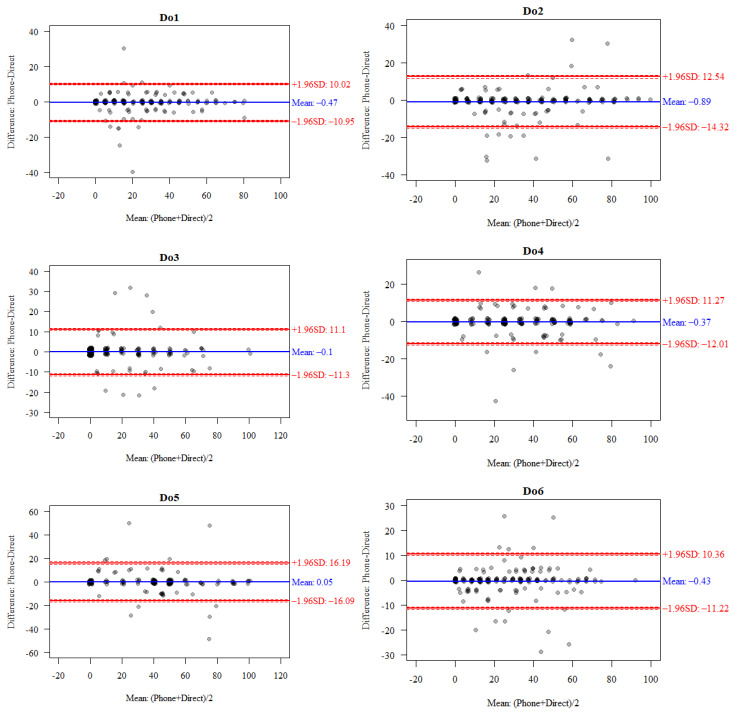
Bland–Altman plot comparing the results in individual subscales of the 36-item WHODAS 2.0 (excluding the Do5.2 subscale) for face-to-face and telephone interviews.

The consistency of the results obtained in individual subscales was also confirmed using the Bland–Altman method. For four of the six subscales, the percentage of participants whose results fell within the 95% confidence interval was less than 5%, while for two (Do2 and Do6) it slightly exceeded this threshold (Table 5, Figure 3).

## 4. Discussion

In a randomised crossover study aimed at assessing the comparability of the results of the 36-item (excluding the Do5.2 subscale) 2.0 in face-to-face and telephone interviews among older adults, we found that both methods of assessment provide comparable results in measuring functional difficulties and disability levels.

For the 32 items analysed in the WHODAS 2.0 questionnaire, the percentage of consistent responses given by respondents during face-to-face and telephone measurements ranged from 85.71% (item D6.4) to 98.03% (item D3.3), indicating a high level of response repeatability. The values of Cohen’s kappa coefficient, used to assess test–retest reliability for qualitative data, ranged from 0.788 (item D6.4) to 0.947 (item D1.1). According to the classification proposed by Landis and Koch [29], kappa values in the range of 0.61–0.80 indicate good agreement, while values from 0.81 to 1.00 suggest very good or almost perfect agreement. In light of these criteria, the results of this analysis confirm the high stability of the WHODAS 2.0 tool in a repeat study using different interview methods—face-to-face and telephone. A comparative analysis of the total scores obtained for 32 questions in the 36-item version of WHODAS 2.0, conducted using two interview methods, showed that the agreement of the assessments was almost perfect for 9 items (κ ≥ 0.81), high for 19 items (0.61 ≤ κ < 0.81), and moderate for 4 items (0.41 ≤ κ < 0.61). These findings indicate a consistently high level of agreement between the two modes of administration, suggesting that the method of conducting the interview had a limited impact on respondents’ answers. However, it should be emphasised that the randomised crossover design used in this study primarily aimed to minimise potential order effects, and no separate analysis was conducted to verify their absence.

The assessment of the consistency of face-to-face and telephone interviews for the total score and subscales of the 36-item WHODAS 2.0 was performed using the intraclass correlation coefficient (ICC). For the total score of the 32-item WHODAS 2.0, the ICC correlation coefficient was 0.986, and for individual domains, it ranged from 0.953 (Do5.1) to 0.967 (Do3). These results indicate a very high consistency of the obtained results.

Similar results were also reported in a study by Moura et al., who assessed the repeatability of the 36-item WHODAS 2.0 in women with urinary incontinence. The first study was conducted in a face-to-face interview, while the retest was conducted by telephone. ICC values exceeding 0.80 were obtained for the total score and ranging from 0.59 to 0.87 for the domains, which also indicates good agreement [30], although lower than in the general population of older adults analysed in our study. Shahedifar et al. did not examine the agreement between face-to-face and telephone survey results but conducted a study of the psychometric properties of the 36-item WHODAS 2.0 using a telephone survey in an Iranian cohort of people with traffic injuries. These authors demonstrated an overall ICC of 0.99 and high ICC values for individual domains (ranging from 0.93 to 0.99), confirming the very good stability of measurements using telephone interviews [31]. Cardoso da Silva et al. conducted a video interview to assess the validity and reliability of the WHODAS 2.0 questionnaire in people who had suffered a stroke. The authors indicated adequate validity and internal consistency (ICC = 0.88; Cronbach’s alpha = 0.88). Discriminant validity showed satisfactory accuracy in distinguishing levels of disability using video conferencing (AUC = 0.67; *p* = 0.04) [32]. The researchers recommended further studies of WHODAS 2.0 using different data collection methods [31].

In this study, the consistency of the total score and the scores in the six subscales analysed in the WHODAS 2.0 was additionally confirmed using the Bland–Altman method. Only the total scores of 5.42% of respondents fell outside the 95% confidence interval of −6.81 to 5.97 points. The results obtained for four of the six subscales analysed fell within the 95% confidence interval, while for domains Do2 and Do6 they slightly exceeded this range.

This study extends the results of previous studies that compared telephone and face-to-face interviews of other standardised questionnaires to assess functional or cognitive impairment in older adults using other scales and found that telephone interviews provide an adequate method of data collection [33,34,35,36]. Some of these studies involved small sample sizes and often used correlation to compare different modes of assessment rather than assessing the degree of agreement, which, according to Dauphinot et al., is a more appropriate methodology for achieving the objective [35]. Although telemedicine diagnostics are not intended to completely replace the gold standard of face-to-face assessment, when used appropriately, they can expand the scope of practice, especially when barriers to standard clinical assessment arise [37].

Telemedicine offers the potential to expand access to healthcare for vulnerable older adults. Although it cannot replace traditional face-to-face examinations, it may serve as a valuable complement to in-person care, particularly for preliminary or screening assessments. The use of standardised, validated tools is essential to ensure the reliability of telemedicine-based evaluations and to enable consistent, evidence-based decision-making.

### Strengths and Weaknesses

To our knowledge, this is the first study comparing face-to-face and telephone interviews using the 36-item WHODAS 2.0 questionnaire (excluding subscale Do5.2). The use of a randomised crossover design minimised the potential impact of interview order (AB/BA) on the results. Although this design helps to balance possible order effects, no separate analysis comparing the results between the two sequences was performed, so the results should be interpreted as controlling for, but not excluding, the influence of this factor. The study was supplemented by an analysis of adverse events that may have occurred between measurements.

However, several important limitations should be considered. First, the participants came from a single region of Poland (south-eastern), which may to some extent limit the generalisability of the results to the national population or other cultural groups. In addition, people with more severe disabilities may have been underrepresented due to the inclusion criterion requiring normal cognitive function (AMTS > 6). Conducting telephone interviews with people with moderate or severe cognitive impairment could lead to results of limited reliability.

It is also worth noting the specific nature of telephone interviews, in which the lack of eye contact limits the possibility of direct observation of elements such as motor functions, facial expressions and non-verbal communication. In face-to-face studies, these factors may provide additional information about functional status, but in our study, the absence of these elements did not affect the consistency of the results. The data obtained indicate that the telephone method can be a reliable and comparable alternative to traditional face-to-face interviews in assessing the functioning of older people.

It should also be noted that the preferences and individual capabilities of participants—such as hearing, familiarity with technology or comfort with remote communication—may vary in the older adult population and potentially affect the level of engagement or completeness of data. However, in this study, no differences were observed that would suggest a significant impact of these factors on the results obtained, confirming that both the telephone and face-to-face formats are well accepted by the respondents.

Apart from the potential limitations indicated, the study design—including random assignment, a standardised procedure and control of confounding factors—was conducted in accordance with COSMIN recommendations, minimising the risk of other significant methodological limitations.

## 5. Conclusions

The results of this randomised crossover study indicate that both face-to-face and telephone interviews using the 36-item WHODAS 2.0 (excluding the Do5.2 subscale) provide comparable and reliable information on the level of functioning of older adults. The high level of response consistency, confirmed by the kappa coefficient, ICC and Bland–Altman analysis, demonstrates the stability of the tool regardless of the form of the interview. The use of the telephone method can therefore be a valuable and practical alternative for screening, especially in situations where access to in-person care is limited. This allows for the expansion of diagnostic capabilities and monitoring of the functioning of the older adult population while maintaining a high level of standardisation and data reliability. Future studies should assess whether regional, cultural or socio-economic factors influence the reliability of telephone interviews and how different forms of communication (telephone, online, face-to-face) affect response rates and data completeness. The reliability of telephone methods in other age groups and in populations with different diseases and levels of disability should also be verified. An analysis of participant preferences and communication dynamics may further help to optimise the use of the WHODAS 2.0 questionnaire and similar tools in clinical practice and telemedicine.

## Figures and Tables

**Table 1:** Sociodemographic characteristics of the study group.

Parameter		Total (*n* = 203)	G1 (*n*1 = 105)	G2 (*n*2 = 98)	*p*-Value
Age [years]	Mean (SD)	73.48 (6.95)	73.65 (7.18)	73.31 (6.74)	0.786 ^(a)^
	Median (quartiles)	72 (68–78)	72 (68–78)	72 (68–78)	
	Range	62–95	62–95	65–93	
Sex	Female	125 (61.58%)	65 (61.90%)	60 (61.22%)	0.921 ^(b)^
	Male	78 (38.42%)	40 (38.10%)	38 (38.78%)	
The Abbreviated Mental Test Score	Mean (SD)	9.28 (0.99)	9.20 (1.07)	9.37 (0.89)	0.446 ^(a)^
	Median (quartiles)	10 (9–10)	10 (9–10)	10 (9–10)	
	Range	7–10	7–10	7–10	
Marital status	In a relationship	106 (52.22%)	53 (50.48%)	53 (54.08%)	0.607 ^(b)^
	Single	97 (47.78%)	52 (49.52%)	45 (45.92%)	
Professional activity	Inactive	185 (91.13%)	12 (11.43%)	6 (6.12%)	0.279 ^(c)^
	Active	18 (8.87%)	93 (88.57%)	92 (93.88%)	
Place of residence	Urban area	84 (41.38%)	59 (56.19%)	60 (61.22%)	0.467 ^(b)^
	Rural area	119 (58.62%)	46 (43.81%)	38 (38.78%)	
Number of people in a household	Mean (SD)	3.23 (1.60)	3.17 (1.60)	3.30 (1.60)	0.550 ^(a)^
	Median (quartiles)	3 (2–4)	3 (2–4)	3 (2–4)	
	Range	1–7	1–7	1–7	
Education level	Vocational or lower	97 (47.78%)	51 (48.57%)	46 (46.94%)	0.957 ^(b)^
	Secondary	56 (27.59%)	29 (27.62%)	27 (27.55%)	
	Higher	50 (24.63%)	25 (23.81%)	25 (25.51%)	
Number of chronic diseases	Mean (SD)	4.94 (2.92)	5.32 (3.22)	4.52 (2.52)	0.102 ^(a)^
	Median (quartiles)	5 (3–6)	5 (3–7)	4 (3–6)	
	Range	0–18	0–18	0–15	
Use of mobility aids	No	150 (73.89%)	73 (69.52%)	77 (78.57%)	0.142 ^(b)^
	Yes	53 (26.11%)	32 (30.48%)	21 (21.43%)	
Number of medications taken daily	4 or more	96 (47.29%)	57 (54.29%)	39 (39.80%)	0.100 ^(b)^
	3	45 (22.17%)	17 (16.19%)	28 (28.57%)	
	2	38 (18.72%)	17 (16.19%)	21 (21.43%)	
	1	17 (8.37%)	11 (10.48%)	6 (6.12%)	
	0	7 (3.45%)	3 (2.86%)	4 (4.08%)	
Pain intensity in last 30 days	Mean (SD)	3.76 (2.16)	3.94 (2.19)	3.56 (2.11)	0.264 ^(a)^
	Median (quartiles)	4 (2–5)	4 (3–5)	3.5 (2–5)	
	Range	0–10	0–10	0–10	
Physical activity	Never	106 (52.22%)	58 (55.24%)	48 (48.98%)	0.616 ^(b)^
	1–2 times a week	70 (34.48%)	33 (31.43%)	37 (37.76%)	
	3 or more times a week	27 (13.30%)	14 (13.33%)	13 (13.27%)	
Quality of life in last 14 days self-assessment	Poor	16 (7.88%)	10 (9.52%)	6 (6.12%)	0.197 ^(b)^
	Neither good nor bad	63 (31.03%)	38 (36.19%)	25 (25.51%)	
	Good	106 (52.22%)	50 (47.62%)	56 (57.14%)	
	Very good	18 (8.87%)	7 (6.67%)	11 (11.22%)	

^(a)^ Mann–Whitney U test. ^(b)^ Chi-square test. ^(c)^ Pearson’s maximum likelihood chi-square test.

**Table 2:** Assessment of consistency between face-to-face and telephone interviews for the 36-item WHODAS 2.0 questions.

Parameter	Agreement	κ	95% CI		Agreement (McHugh)
D1.1	96.55%	0.947	0.908	0.986	Almost perfect
D1.2	91.13%	0.873	0.817	0.929	Strong
D1.3	92.61%	0.888	0.834	0.943	Strong
D1.4	89.16%	0.849	0.788	0.909	Strong
D1.5	91.63%	0.795	0.706	0.884	Moderate
D1.6	90.64%	0.792	0.706	0.879	Moderate
D2.1	92.61%	0.906	0.860	0.952	Almost perfect
D2.2	92.12%	0.870	0.810	0.930	Strong
D2.3	92.61%	0.846	0.771	0.920	Strong
D2.4	89.66%	0.833	0.768	0.898	Strong
D2.5	88.67%	0.858	0.803	0.913	Strong
D3.1	93.10%	0.877	0.816	0.938	Strong
D3.2	94.09%	0.866	0.796	0.937	Strong
D3.3	98.03%	0.931	0.864	0.998	Almost perfect
D3.4	89.66%	0.834	0.769	0.900	Strong
D4.1	95.07%	0.926	0.882	0.970	Almost perfect
D4.2	94.58%	0.905	0.850	0.959	Almost perfect
D4.3	94.58%	0.892	0.831	0.952	Strong
D4.4	91.63%	0.877	0.821	0.933	Strong
D4.5	86.70%	0.827	0.767	0.887	Strong
D5.1	93.60%	0.909	0.861	0.956	Almost perfect
D5.2	93.10%	0.904	0.856	0.952	Almost perfect
D5.3	88.67%	0.845	0.785	0.905	Strong
D5.4	91.63%	0.888	0.837	0.939	Strong
D6.1	94.09%	0.914	0.867	0.961	Almost perfect
D6.2	94.09%	0.909	0.859	0.959	Almost perfect
D6.3	91.63%	0.856	0.792	0.921	Strong
D6.4	85.71%	0.788	0.718	0.859	Moderate
D6.5	87.68%	0.824	0.759	0.888	Strong
D6.6	89.16%	0.842	0.780	0.904	Strong
D6.7	87.19%	0.794	0.722	0.865	Moderate
D6.8	89.66%	0.842	0.780	0.905	Strong

**Table 3:** Assessment of the consistency of face-to-face interviews with telephone interviews for the total score of the 32-item WHODAS 2.0.

ICC	95% CI		Agreement (Koo & Li)				
0.986	0.982	0.989	Excellent				
**Bland–Altman**							
**WHODAS 2.0 total (Mean±SD)**			**RC**	**VC**	**LOA (95% CI)**		**Bland–Altman** **index**
**Direct**	**Phone**	**Difference:** **Direct-Phone**			**Lower** **limit**	**Upper** **limit**	
27.3 ± 19.44	26.87 ± 19.52	−0.42 ± 3.26	6.52	12.03%	−6.81 (−6.99; −6.63)	5.97 (5.79; 6.14)	5.42%

RC—repeatability coefficient; VC—variation coefficient; LOA—limits of agreement.

**Table 4:** Assessment of the consistency of face-to-face and telephone interviews in individual subscales using the intraclass correlation coefficient.

Subscale	ICC	95% CI		Agreement (Koo & Li)
Do1	0.964	0.953	0.973	Excellent
Do2	0.964	0.953	0.973	Excellent
Do3	0.967	0.956	0.975	Excellent
Do4	0.966	0.955	0.974	Excellent
Do5.1	0.953	0.939	0.964	Excellent
Do6	0.964	0.953	0.972	Excellent

**Table 5:** Assessment of the consistency of face-to-face and telephone interviews in individual subscales using the Bland–Altman method.

Subscale	Total (Mean ± SD)			RC	VC	LOA (95% CI)		Bland–Altman Index
	Direct	Phone	Difference: Direct-Phone			Lower Limit	Upper Limit	
Do1	23.65 ± 19.88	23.18 ± 20.02	−0.47 ± 5.35	10.7	22.85	−10.95 (−11.43; −10.47)	10.02 (9.54; 10.5)	3.45%
Do2	34.27 ± 25.37	33.37 ± 25.87	−0.89 ± 6.85	13.71	20.26	−14.32 (−15.11; −13.53)	12.54 (11.75; 13.33)	5.42%
Do3	16.4 ± 22.26	16.31 ± 22.02	−0.1 ± 5.72	11.43	34.95	−11.3 (−11.85; −10.75)	11.1 (10.55; 11.65)	3.94%
Do4	28.57 ± 22.89	28.2 ± 22.54	−0.37 ± 5.94	11.88	20.92	−12.01 (−12.6; −11.42)	11.27 (10.68; 11.86)	4.43%
Do5.1	36.8 ± 27.31	36.85 ± 26.42	0.05 ± 8.24	16.47	22.36	−16.09 (−17.23; −14.95)	16.19 (15.05; 17.33)	4.93%
Do6	25.64 ± 20.49	25.21 ± 20.49	−0.43 ± 5.51	11.01	21.66	−11.22 (−11.73; −10.71)	10.36 (9.85; 10.87)	6.40%

RC—repeatability coefficient; VC—variation coefficient; LOA—limits of agreement.

## Data Availability

The raw data supporting the findings of this study are available in an open repository at the following link: https://rdb.ur.edu.pl/items/0f982b1b-b617-4371-9f72-f7f7352538ab. The data presented in this study are also available on reasonable request from the corresponding author.
